# Supplementary material for: On Hallucinations in Tomographic Image Reconstruction
Source: IEEE Trans Med Imaging. Author manuscript; Available in PMC 2021 Dec 15. (PMC8673588; doi:10.1109/TMI.2021.3077857)
Supplement: supp1-3077857 [file NIHMS1751927-supplement-supp1-3077857.pdf]

On hallucinations in tomographic image reconstruction (Supplementary Material)

Sayantan Bhadra, Varun A. Kelkar, Frank J. Brooks and Mark A. Anastasio, *Senior Member, IEEE*

S. I. Sampling mask

A uniform Cartesian undersampling mask was employed in our simulation studies for generating the incomplete k-space data described in Sec. IV-A of the manuscript. The full k-space data were undersampled by a factor of 3 using the uniform sampling mask shown in Fig. S. 1.

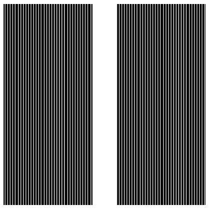

Fig. S. 1: Sampling mask

S. II. Details of reconstruction methods

The details of the three reconstruction methods employed in our studies are presented below. They are based on the imaging model:

$$\mathbf{g} = \mathbf{H}\boldsymbol{\theta} + \mathbf{n}, \tag{1}$$

where $\boldsymbol{\theta} \in \mathbb{E}^N$ is the sought-after coefficient vector, $\mathbf{g} \in \mathbb{E}^M$ is the observed measurement data, $\mathbf{H} \in \mathbb{E}^{M\times N}$ is the system matrix, and $\mathbf{n} \in \mathbb{E}^N$ is iid Gaussian noise.

A. Penalized least squares with TV regularization (PLS-TV)

The PLS-TV method involves solving the penalized least-squares optimization framework with the penalty term as the TV penalty:

$$\hat{\boldsymbol{\theta}} = \underset{\boldsymbol{\theta}}{\operatorname{argmin}} \, ||\mathbf{g} - \mathbf{H}\boldsymbol{\theta}||_2^2 + \lambda ||\boldsymbol{\theta}||_{TV}, \tag{2}$$

where λ is the regularization parameter. Proximal gradient methods are commonly employed to implement the PLS-TV method [1]–[3]. In this study, PLS-TV reconstruction was performed for a dataset of measurements corresponding to 69 different images using the Berkeley Advanced Reconstruction Toolbox (BART) [4], [5]. BART performs PLS-TV reconstruction using the augmented Lagrangian based optimization method proposed in [3]. The regularization parameter λ in Eq. (2) was chosen by first performing image reconstruction on a subset of the dataset, with different values of λ. The value of λ which provided the lowest mean of the root mean squared error (RMSE) metric over the subset was chosen, and used for image reconstruction of all the images in the dataset.

B. U-Net-based reconstruction

The following procedure, known as *image-domain learning*, was employed for the U-Net based image reconstruction. First, initial estimates of the images were obtained from the measurement data by use of the pseudoinverse operator. They were then employed as inputs to a convolutional neural network (CNN), which was trained in order to produce artifact-free images, similar to images from the ground truth distribution [6]–[9]. As is common practice, the CNN architecture used in this study is the U-Net [10]. A U-Net consists of two CNNs that represent a downsampling path followed by an upsampling path respectively, and skip connections [11] between similar levels in the downsampling and upsampling paths. Let the initial estimate from the measurement data be denoted as $\boldsymbol{\theta}'$ and the function computed by the U-Net be represented as $B(\boldsymbol{\theta}'; w)$ where $B : \mathbb{E}^N \to \mathbb{E}^N$ and w denotes the weight parameters of the U-Net. Given a training data set of initial estimate-ground truth pairs $\left\{\boldsymbol{\theta}_i', \boldsymbol{\theta}_i\right\}_{i=1}^D$ where D is the size of the training data set, the optimal weight parameters w^* are learned by approximately solving the following optimization problem:

$$w^* = \underset{w}{\operatorname{argmin}} \sum_{i=1}^{D} \mathcal{L}\Big(B(\boldsymbol{\theta}_i', w), \boldsymbol{\theta}_i\Big), \tag{3}$$

where $\mathcal{L}(\cdot,\cdot)$ is a suitable loss function. In this work, mean absolute error was used as the loss function [12]. The model for the U-Net was based on the single-coil baseline U-Net architecture provided in [13]. A stochastic gradient-based method known as RMSProp [14] was employed to solve the optimization problem in Eq. (3). After this iterative scheme for training the U-Net reached convergence, the trained U-Net was used to reconstruct images from a previously unseen test measurement dataset, where an initial estimate $\boldsymbol{\theta}'_{test}$ computed from a test measurement data was employed to obtain the reconstructed image $\hat{\boldsymbol{\theta}}_{test} = B(\boldsymbol{\theta}'_{test}, w)$. The training and testing of the U-Net based reconstruction was performed using code available at https://github.com/facebookresearch/fastMRI, which utilizes PyTorch Lightning [15].

C. Deep image prior (DIP)

Recently, Ulyanov *et al.* [16] showed that a CNN $G : \mathbb{R}^k \to \mathbb{E}^N$ with randomly initialized weights w and random input $\mathbf{z} \in \mathbb{R}^k$ can be an effective regularizer for image restoration problems such as denoising, super-resolution and

inpainting. This method of regularization, known as deep image prior (DIP), utilizes the observation that the structure of deep convolutional networks captures several low-level image statistics and is biased towards smooth, natural images. Van Veen *et al.* [17] extended the DIP framework to applications in tomographic imaging from incomplete measurements with encouraging results. Essentially, image reconstruction using the DIP method can be formulated in terms of the following optimization problem:

$$w^* = \underset{w}{\text{argmin}}\, ||\mathbf{g} - \mathbf{H}G(\mathbf{z}; w)||_2^2, \quad \hat{\boldsymbol{\theta}} = G(\mathbf{z}; w^*) \tag{4}$$

where $\mathbf{z}$ and w are randomly initialized.

It has been shown in [16], [17] that the DIP method overfits the measurement noise upon convergence. Hence, further regularization may be required, either in the form of early stopping or with the addition of penalties in the optimization problem in Eq. (4). Inspired by [18], in our experiments, image reconstruction using the DIP method with TV regularization (DIP-TV) was performed by approximately solving the following optimization problem:

$$w^* = \underset{w}{\text{argmin}}\, ||\mathbf{g} - \mathbf{H}G(\mathbf{z}, w)||_2^2 + \lambda||G(\mathbf{z}, w)||_{\text{TV}}, \quad \hat{\boldsymbol{\theta}} = G(\mathbf{z}; w^*) \tag{5}$$

where $\mathbf{z}$ and w were randomly initialized, and λ is the regularization parameter. The same U-Net architecture employed for the U-Net based reconstruction was employed for DIP-TV, and was implemented in TensorFlow [19]. Similar to the implementation of the PLS-TV method as outlined in Sec. S. II-A, the regularization parameter λ for the TV penalty in Eq. (5) was chosen by first performing image reconstruction on a subset of the dataset, with different values of λ. Subsequently, the value which provided the lowest mean RMSE over the subset was chosen to perform image reconstruction from all the measurements. The optimization problem in Eq. (5) was approximately solved using a stochastic gradient algorithm called Adam [20].

S. III. Examples of Measurement Space Hallucination Maps

While the results in our simulation studies focused on the effect of null space hallucination maps, measurement space hallucination maps can also be computed corresponding to reconstructed images from different methods. The measurement space hallucination map is denoted as

$$\hat{\boldsymbol{\theta}}^{HM}_{meas} \equiv \hat{\boldsymbol{\theta}}_{meas} - \hat{\boldsymbol{\theta}}_{tp}, \tag{6}$$

which describes the consistency between the measurement component of the reconstructed image $\hat{\boldsymbol{\theta}}_{meas}$ with respect to the truncated pseudoinverse solution $\hat{\boldsymbol{\theta}}_{tp}$ that can be stably obtained from the measurement data $\mathbf{g}$. Furthermore, unlike the null space hallucination map, the computation of $\hat{\boldsymbol{\theta}}^{HM}_{meas}$ does not require the knowledge of the true object $\boldsymbol{\theta}$. On the other hand, the error map between $\hat{\boldsymbol{\theta}}_{meas}$ and $\boldsymbol{\theta}_{meas}$ is a similar but different error quantity that lies in the measurement space $\mathcal{N}^{\perp}(\mathbf{H})$ and requires the knowledge of $\boldsymbol{\theta}$:

$$\hat{\boldsymbol{\theta}}^{EM}_{meas} \equiv \hat{\boldsymbol{\theta}}_{meas} - \boldsymbol{\theta}_{meas}. \tag{7}$$

In some cases, $\hat{\boldsymbol{\theta}}^{HM}_{meas}$ and $\hat{\boldsymbol{\theta}}^{EM}_{meas}$ may not convey the same information due to the differences that can exist between $\boldsymbol{\theta}_{meas}$ and $\hat{\boldsymbol{\theta}}_{tp}$. These differences arise when there is significant measurement noise in the imaging system or due to modeling error in $\mathbf{H}$, or both. With the "true" imaging operator denoted as $\tilde{\mathbf{H}}$ and the assumed imaging operator as $\mathbf{H}$, Eq. (1) can be re-written as

$$\mathbf{g} = \tilde{\mathbf{H}}\boldsymbol{\theta} + \mathbf{n}. \tag{8}$$

Accordingly, $\hat{\boldsymbol{\theta}}_{tp}$ can be expressed as

$$\hat{\boldsymbol{\theta}}_{tp} = \mathbf{H}^+_P\mathbf{g} \approx \mathbf{H}^+_P(\tilde{\mathbf{H}}\boldsymbol{\theta} + \mathbf{n}) = \mathbf{H}^+_P\tilde{\mathbf{H}}\boldsymbol{\theta} + \mathbf{H}^+_P\mathbf{n}. \tag{9}$$

On the other hand, $\boldsymbol{\theta}_{meas}$ is represented as

$$\boldsymbol{\theta}_{meas} \equiv \mathbf{H}^+_P\mathbf{H}\boldsymbol{\theta}. \tag{10}$$

It can be observed from Eq. (9) and Eq. (10) that, when either or both of the quantities $||\mathbf{n}||_2^2$ and $||\mathbf{H} - \tilde{\mathbf{H}}||_2^2$ is non-trivial, $||\hat{\boldsymbol{\theta}}_{tp} - \boldsymbol{\theta}_{meas}||_2^2$ is likely to be significant. In such cases, $\hat{\boldsymbol{\theta}}^{HM}_{meas}$ and $\hat{\boldsymbol{\theta}}^{EM}_{meas}$ may represent different information.

Figure S.2 shows examples of measurement space hallucination maps for images reconstructed by the U-Net method corresponding to an in-distribution (IND) object and an out-of-distribution (OOD) object. It can be observed that the measurement component error map has appreciable differences compared to the measurement space hallucination map. These differences can be attributed to the presence of non-trivial measurement noise as well as additional phase noise disturbance during simulation of the k-space data resulting in model error, since the phase noise is unknown and assumed to be zero during reconstruction. The measurement space hallucination maps as shown here may provide an insight into how well a given reconstruction method maintains data consistency and be compared with other reconstruction methods.

References

[1] A. Chambolle, "An algorithm for total variation minimization and applications," *Journal of Mathematical Imaging and Vision*, vol. 20, no. 1-2, pp. 89–97, 2004.

[2] A. Beck and M. Teboulle, "Fast gradient-based algorithms for constrained total variation image denoising and deblurring problems," *IEEE Transactions on Image Processing*, vol. 18, no. 11, pp. 2419–2434, 2009.

[3] M. V. Afonso, J. M. Bioucas-Dias, and M. A. Figueiredo, "An augmented lagrangian approach to the constrained optimization formulation of imaging inverse problems," *IEEE Transactions on Image Processing*, vol. 20, no. 3, pp. 681–695, 2010.

[4] M. Uecker, F. Ong, J. I. Tamir, D. Bahri, P. Virtue, J. Y. Cheng, T. Zhang, and M. Lustig, "Berkeley advanced reconstruction toolbox," in *Proc. Intl. Soc. Mag. Reson. Med*, vol. 23, no. 2486, 2015.

[5] M. U. *et al.*, "Bart toolbox for computational magnetic resonance imaging, doi: 10.5281/zenodo.592960," 2015. [Online]. Available: http://mrirecon.github.io/bart/

[6] K. H. Jin, M. T. McCann, E. Froustey, and M. Unser, "Deep convolutional neural network for inverse problems in imaging," *IEEE Transactions on Image Processing*, vol. 26, no. 9, pp. 4509–4522, 2017.

[7] Y. Han and J. C. Ye, "Framing U-Net via deep convolutional framelets: Application to sparse-view CT," *IEEE transactions on medical imaging*, vol. 37, no. 6, pp. 1418–1429, 2018.

[8] C. M. Hyun, H. P. Kim, S. M. Lee, S. Lee, and J. K. Seo, "Deep learning for undersampled MRI reconstruction," *Physics in Medicine & Biology*, vol. 63, no. 13, p. 135007, 2018.

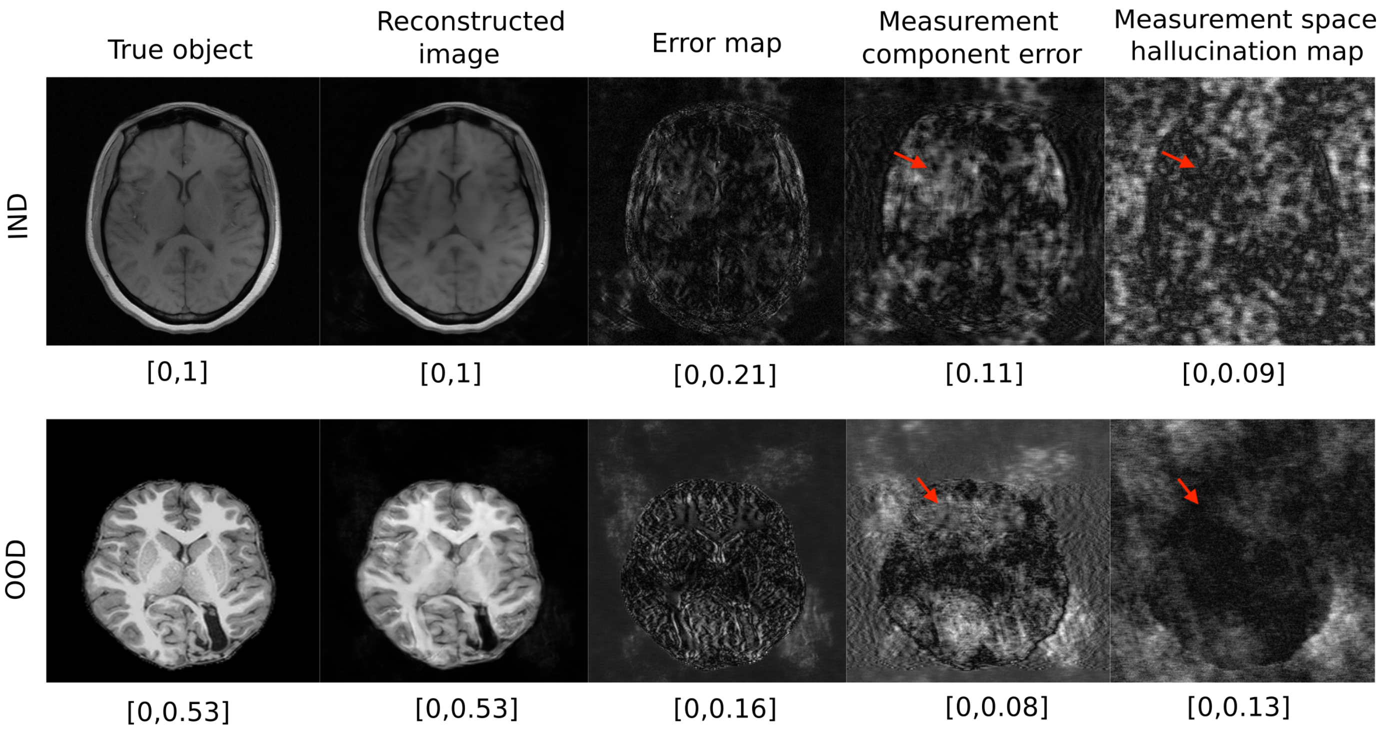

Fig. S. 2: Measurement space hallucination maps for reconstructed images using the U-Net method corresponding to an IND (above) and an OOD (below) object. Note that the measurement component error map and the measurement space hallucination map are appreciably different. The red arrows point towards a region in each type of object where such differences can be clearly seen.

[9] S. Antholzer, M. Haltmeier, and J. Schwab, "Deep learning for photoacoustic tomography from sparse data," *Inverse problems in science and engineering*, vol. 27, no. 7, pp. 987–1005, 2019.

[10] O. Ronneberger, P. Fischer, and T. Brox, "U-Net: Convolutional networks for biomedical image segmentation," in *Medical Image Computing and Computer-Assisted Intervention – MICCAI 2015*, N. Navab, J. Hornegger, W. M. Wells, and A. F. Frangi, Eds. Cham: Springer International Publishing, 2015, pp. 234–241.

[11] M. Drozdzal, E. Vorontsov, G. Chartrand, S. Kadoury, and C. Pal, "The importance of skip connections in biomedical image segmentation," in *Deep Learning and Data Labeling for Medical Applications*. Springer, 2016, pp. 179–187.

[12] H. Zhao, O. Gallo, I. Frosio, and J. Kautz, "Loss functions for image restoration with neural networks," *IEEE Transactions on computational imaging*, vol. 3, no. 1, pp. 47–57, 2016.

[13] J. Zbontar, F. Knoll, A. Sriram, M. J. Muckley, M. Bruno, A. Defazio, M. Parente, K. J. Geras, J. Katsnelson, H. Chandarana *et al.*, "fastMRI: An open dataset and benchmarks for accelerated MRI," *arXiv preprint arXiv:1811.08839*, 2018.

[14] T. Tieleman and G. Hinton, "Lecture 6.5—RMSProp: Divide the gradient by a running average of its recent magnitude," COURSERA: Neural Networks for Machine Learning, 2012.

[15] W. Falcon, "Pytorch lightning," *GitHub. Note: https://github.com/PyTorchLightning/pytorch-lightning Cited by*, vol. 3, 2019.

[16] D. Ulyanov, A. Vedaldi, and V. Lempitsky, "Deep image prior," in *Proceedings of the IEEE Conference on Computer Vision and Pattern Recognition*, 2018, pp. 9446–9454.

[17] D. Van Veen, A. Jalal, M. Soltanolkotabi, E. Price, S. Vishwanath, and A. G. Dimakis, "Compressed sensing with deep image prior and learned regularization," *arXiv preprint arXiv:1806.06438*, 2018.

[18] J. Liu, Y. Sun, X. Xu, and U. S. Kamilov, "Image restoration using total variation regularized deep image prior," in *ICASSP 2019-2019 IEEE International Conference on Acoustics, Speech and Signal Processing (ICASSP)*. IEEE, 2019, pp. 7715–7719.

[19] M. Abadi, P. Barham, J. Chen, Z. Chen, A. Davis, J. Dean, M. Devin, S. Ghemawat, G. Irving, M. Isard *et al.*, "Tensorflow: a system for large-scale machine learning." in *OSDI*, vol. 16, 2016, pp. 265–283.

[20] D. P. Kingma and J. Ba, "Adam: A method for stochastic optimization," *arXiv preprint arXiv:1412.6980*, 2014.
